# Cryptogamous Origin of Fevers and Other Diseases

**Published:** 1866-11

**Authors:** J. M. Johnson

**Affiliations:** Atlanta, Ga.


ATLANTA

### Medical and Surgical Journal.

NEW SERIES.

VOL. VIIB NOVEMBER, 1866. No. 9.

### ORIGINAL COMMUNICATIONS.

### ARTICLE I.

*Cryptogamous origin of Fevers and other Diseases.* By J. M. JOHNSON, M. D., Atlanta, Ga.

The overwhelming importance of this subject commends it to the attention of thinking men everywhere. There is not, in the world, a conscientious physician that has not flt humiliated at the multitude of discordant opinions as to the cause or causes of fever. Men of all capacities, and from various motives, have appeared before the public in the roll of advocates and objectors to the various theories of the day, and have only left confusion doubly confounded, to mark the ground over which they have blundered. The substantial benefit, since the days of Lancisi, in 1695, may be summed up in one word: nothing. Whilst the literature of medicine has been almost hopelessly encumbered with high sounding latin verbs and adjectives, putting it out of the power of earnest men, constantly laboring at the bed-side, to keep up with their newly coined names and phrases, too often a cloak for poor opinions. There is everywhere a class of sound, practical, original thinkers, who scarcely leave the field of their labors half dozen months in a life-time, who are practicing medicine with good success, but who never give to the world the benefit of their experience, rich in observation, in thought, and especially in results, because they are unaccustomed to the use of the pen, and can not measure terms with a majority of contributors to the various journals.

Amongst this class there are many who have been earnest inquirers into the mysteries of causation, and are to-day laden with rich fruit, which the reasons I have enumerated have prevented being given to the world. A good economist would husband his resources better than this. The farmer applies the waste of his house and barn yard to the failing spots in his field. The financier will not spend his cash in bank, but gather up the odds and ends with which to keep up his household equipage. In violation of this sound policy, shall we commit the true interests and true literature of the time-honored profession of medicine, to wordy authors who write for notoriety, and who deal in hyperbole to cover up a weak argument, or conceal a want of knowledge? Quackery is rampant from one end of the country to the other. These bold *guerillas* derive much of their consequence from the heterogenious mass of opinions, so patent to everybody. Not only in the public journals, but in every neighborhood, where you find two doctors, they seem to make it a point, publicly, to disagree in some of the important details of medicine. This results, not so much from a disposition to play the charlatan, as from the vast contrariety of opinions promulgated under the sanction of great names.

There is a universally known vulgarism—we hear it every day—and alas! it is too true, that "doctors will differ."

The true dignity and usefulness of the profession will not be reached until there is a boundary fixed to dissention and the endless conflict of opinion. This is not impossible. Suppose we were to memorialize Congress for an appropriation of two hundred thousand dollars per annum, to be applied to the payment of the salaries and expenses of a board of scientific and practical men, to be appointed by the medical societies of the several States, during the term of each Presidential Administration, whose duty it would be to visit every part of the United States, and make such tests and experiments as would enable them to report upon the medical topography of the whole country; make it their province, also, to report upon all new discoveries, to correspond with and visit active practitioners in the different localities, and collect such medical statistics as will enable them to report upon the true nature and treatment of all of the diseases of the country.

There are in the United States more than fifty thousand physicians: many of them men of eminent talents, social position, and influence. The whole land is interested in medical reform and progress. Not a man, woman, or child but has a deep interest in, and all are dependent upon, and look up to their medical advisers in the trying emergencies of disease and death; and surely an appeal from such a body of men could not be made in vain, to the magnanimity of Congress. Look at the profession of the law: there is not a session of Congress, or of the Legislature of any of the States, that does not enact laws or appropriate money to codify the statutes, and publish the decisions of Courts and acts of their various bodies. Is money and land of more value than health and life? The profession of medicine does as much to promote the great cause of social progress as any of the learned professions, hardly excepting that of the gospel of peace; and in its appropriate sphere, where disease and death visit the hearthstone, and friends are powerless to help, then it is that the faithful physician, with steady nerves and cheerful counsel, dispels alarm, and opens up the boon of hope to the fainting soul.

I have said this much as introductory to the subject. The interesting paper lately published by Dr. Salisbury, in the Philadelphia *Journal of Medical Sciences* for January last, but which did not fall under my notice until a day or two ago, puts a new face upon an old subject. As far back as 1695, Lancisi published a treatise, "*de noxiis paludum effluviis*," which was, so far as known, the first published theory upon the subject of the production of fever by marsh air, although allusion had been made to the subject by some of the oldest authors. Suqsequently, McCullough, of London, and Cragie, of Edinburgh, gave it the influence of their powerful names, and it became the accepted theory of causation everywhere. But the great intangibility of the subject, growing out of the imponderableness of the agent, so baffled inquiry, as to its real character, that finally grave doubts had arisen as to the verity of a theory universally accepted for more than a century and a half, as a finality of the causation of intermittant and other fevers; and, indeed, it is full of difficulties, even admitting the correctness of Dr. Salisbury's paper, to the full extent of his own credulity. Why are not all paludal localities visited by the same endemic and epidemic distempers? In treating upon this subject, I shall depend upon many facts furnished by the late Dr. John Kearnsey Mitchell, of Philadelphia, whose brilliant pen and benevolent nature have erected a monument to his memory, which I fondly hope will out-live the neglect of friends, or the fame of the "merry andrews" who may attempt to appropriate his opinions and labors.

The hypothesis of the miasmatists, that there was exhaled from swampy low-grounds an organic poison which produced intermittent and remittent fevers, has, by the use of the microscope in the hands of Dr. Salisbury, been demonstrated. Not only does he find the very agent, but gives a description of the plant, and the abnormal bodies exhaled from it, and which, from their minuteness, are capable of absorption by the skin; are received into the lungs by inhalation; swallowed with food and water; organized with the ingesta, and diffused by means of the circulation, poisoning the epithelial surfaces with which they come in contact,—and thus working the morbid changes upon the blood, the nervous centres, and trunks, spleen, liver, kidneys, and skin, so frequently observed amongst those inhabiting ague districts, especially those who are the subjects of ague paroxysms.

In the following pages I shall quote extensively from Dr. Salisbury's paper, and on important points give his own words.

The field of Dr. Salisbury's late discoveries is by no means new. In every country where the arts and sciences have been developed and cultivated, descriptions of cryptogamous plants abound, showing their diffusion to be *pari passu* with the universe, and their number and variety almost equaling the firmament of stars, or the sands of the sea. Their poisonous properties, and their peculiar seasons of growth, the minuteness of their spores, their love of darkness, and tainted soils, and heavy atmospheres, are, according to Dr. Mitchell, proverbial. For nearly two centuries past, the populace of many parts of Spain believe that mushrooms cause fever. Leger (1775, Vienna) says a mildew fell after thunder showers, which dried and burnt the grass and leaves, and was poisonous for twenty-four hours after falling. Van Eesenbeck believed that mushrooms of the most minute forms had their origin in the air, producing spots and stains, and were the true cause of epidemics. By the use of the microscope Müller discovered that vegetable cells resemble the primordal cells of our own tissues, and inclines to the opinion that these are the true morbid seed.

Dr. Mitchell says, "The mushroom (*fungi*) is equally distant from plants and animals—mere fortuitous developments of vegeto-animal matter, called together by special conditions, and capable of being propagated under circumstances apparently the most contrasted." * * * Of all vegetables, they are the most highly animalized. Like animals, they disengage carbonic acid, and imbibe a quantity of oxygen, and some even extricate hydrogen and nitrogen. Their chemical composition allies them to animal structures. They yield resin, sugar, fungic acid, and a number of saline compounds, and also adepocire albumen, and osmazome of the animal kingdom. By the proper means, they are made to yield several acids and fatty substances, and even wax, tallow, and pure oil.

The two families of plants are distinguished by the generic names of phanerogamic and cryptogamic. The former have organs of generation by which they propagate their species, as wheat, corn, trees, etc., etc. The latter have no organs of generation, and propagate their species by spores. The former embrace every variety of vegetable life, except the mushroom, lichens, and algea, which are embraced in the generic name cryptogami ; and of these, as I have said, the variety is almost endless. They vary in size, from a mere point, requiring the aid of the microscope, to more than thirty pounds weight. Their increase is magical. From a single spore, on the authority of Carpenter, the physiologist, they may grow in a night to the size of a large gourd, and estimated to contain four thousand five hundred millions of cells.

The spore, let it be borne in mind, is the reproductive element. The plant is an aggregation of cells, answering exactly to the primordal cells which make up our own corpus animi. The spores are many times smaller than the cells ; but both are so small as to require a microscope of great power to see them. Even the algoid plant, (one of the cryptogami) has to be aggregated to be seen with the naked eye. This is also called palmella, by Dr. Salisbury, who, in his classifications and names, has " darkened council by words." His latin verbs and adjectives will read badly anywhere out of America.

To the several forms of this type (palmellæ), he has given the generic name of Gemiasma (earth miasma): each of the type having cells consisting of a thin outside wall, and enclosing an inside cell, filled with minute spores, and enclosed within a parent membrane. They are of all colors. Most of this species (palmellæ of the algoid type) act as malarious poisons. The brick-red, green, and lead color, are principally found upon rich calcarious soils ; while the greenish-yellow, and white, are found mostly upon non-calcarious soils. The red, resembling brick dust, sprinkled over the soil, produces intermittents of a congestive type. In all of these is found minute spores, in almost incomprehensible multitudes, that have escaped from the plants beneath ; and these, the most minute of all known organisms, are elevated at night, with the earth exhalations.

The palmella, or poisonous cryptogam, is the variety to which attention is mainly called. It abounds in bogs and low grounds; but not alone in these. It exists in all alluvial and limestone soils, and in the standing ponds and pools everywhere. It is vulgarly known as green moss, where it appears in ponds or sluggish streams. Its generic name is algea. The poison resides, not alone in the cells, but in the spores also. These being much more minute and imponderable, may pass through the skin, and enter the systemic circulation. It is questionable whether the cells do this; but there is abundant means through the lungs, and in food and water, for them to enter the system. The algea is not particularly noxious, except during the process of dessication. When the water has dried up, leaving the algea, and other aquatic products of a cognate nature, on the muck, and exposed to the hot sun, the process of dessication developes the plant in the new phase I have mentioned, and gives off its spores and cells through the medium of cool humid exhalations from the poison locality, in such immense quantities, as to be seen by the naked eye, having the appearance of mist or fog, and presenting a well defined zone, supported by an atmosphere of greater density than that surrounding and above it. Within this zone is contained the "miasma," "malaria," "morbid agent," "morbific cause," or the "*de noxiis palludum effluviis*" of Lancisi, described more than a century and a half ago by many of the old writers.

This zone, with its accumulated poison, more so if possible, than the fabled pandora's box, may be driven by winds to distant and healthy locations, and produce its legitimate fruits where it has never before been felt. It may rise, according to Dr. Salisbury, from thirty-five feet to more than one hundred, and be driven by winds east, west, north, or south; but as the atmosphere gets dry, and as its coolness and density is lessened, it settles to the ground, and remains innoxious through the day, to be exhaled again at night, and recommence its rotine of mischief.

The insolubrity of the night air has been proverbial in all latitudes. It has been denied by some writers, but affirmed by an overwhelming majority. Dr. Lind, on the authority of Dr. J. K. Mitchell, says that sixteen of the crew of the British sloop, Phœnix, went ashore at the island of St. Thomas, on the coast of Africa, and spent the night, and all but two died of the African fever. The remainder of the crew, two hundred and eighty men, went ashore in the day time, and returned at night to their quarters on ship-board, and not one was even sick, although they went where they pleased, washed their clothes, etc. The same is true of the crew of the Hound, who went ashore every day, had their field sports and amusements, such as fishing, etc., and returned at night to the ship, and not one died from any cause. The same author speaks of a settlement of French protestants in a paludal part of Florida, where most of them died. They were visited by eight gentlemen from a healthier place, who spent one night; every one of these was attacked with intermittant fever, and two died. Seven other gentlemen came the next morning after the first party had arrived, and spent the day only, and left before night, and not one was attacked.

Dr. Hunter, of Jamaica, relates many such cases. In one instance, out of sixty or seventy persons sent ashore for water, and who were exposed to the night air, not one escaped fever, while the rest of the crew enjoyed good health.

Dr. James Johnson remarks, that while cruising, or lying at anchor between Batavia and Malacca, every man who went ashore at night died.

Tratter, speaking of the same coast, says: "Every man who slept ashore died, while the rest of the ship's company remained remarkably healthy." Dr. Evans, writing from the unhealthy island of St. Lucie, says: "The sportsman wades through the stagnant waters and mangrove bushes, which cover the surface of the fens, with impunity, through the day, but places himself out of reach of the poisonous effluvia long before sun-set."

Mr. Webb says the British army was almost annihilated at Walcheren, while those on ship-board and out of the night air, escaped entirely.

Robert Armstrong says the same thing of the West Indies, South American coast, and South Pacific Islands.

Many localities in the Southern States are equally pestilential at night. To sleep one night in certain localities in South Carolina, Florida, Georgia, Alabama, Louisiana, etc., etc., will place in jeopardy the life of any unacclimated person, whilst day visits are unattended with danger.

The Danes, I believe, were the first to require scientific observations and reports from their exploring squadrons. This policy has been adopted by every maritime nation of the earth. There is not a continent or an island that has not been thus visited, and contributed its share to the wonders of this wonderful world. In one thing, at least, all agree: that on the coast of Africa, the West Indies, North and South American, and Indian coast—in short, every continent, and every island that dots the ocean—where malignant endemic fevers prevail, the night alone is surely fatal to the unacclimated constitution.

In vain do we search, says Dr. J. K. Mitchell, "in the works on received theories, for the cause of this curious influence of night. It is in the day-time that evaporation goes on most rapidly, and that chemical changes, produced by both heat and light, are in most active operation. The water is warmer, the common vegetation more vivid, and the great chemist, the sun, is urging on the process of the laboratory of nature. This is, of course, admitted by many writers, some of whom confess manfully this part of their subject, while others suppose that the miasm evolved during the day descends during the night. Were this so, it would scarcely account for the difference in the disease producing power between the night and day." Dr. Mitchell is of opinion, that the devevelopment of cryptogams at night explains the analogy between malaria and these plants.

He says: "It is in vain that we search in the latter part of the day for young mushrooms. It is only in the early morning that they are found in their prime and abundance." "A field at evening, exhibiting not a single plant, is often white with them in the morning."

Comstock says: "They spring up almost everywhere, especially amongst decaying substances, and that thousands of them may be seen in the morning, where none existed the evening before." At what time the water cryptogams grow, can only be judged of by the habits of the cognate varieties we have been considering. Whether the green moss of stagnant pools, (algea, confervia, etc.,) or the algoid plants of calcarous and peaty soils, which are closely allied, grow exclusively at night, like the mushroom, is a question only for the curious, and in no way affects the great question of their agency in the production of fever. As the water evaporates, the algea is left upon the muck to dry. By the time dessication is complete, the algea has disappeared, and the minute palmella takes its place under a new organism, with increased power of propagation and mischief. Both of these, according to Dr. Salisbury, take place at night. The process of fructification, during the period of darkness, is only equalled by the rapid exhalation, through the medium of the cold humid atmosphere of night.

Dr. Mitchell is of opinion, that these plants have electrical relations that aid in their growth and dissemination; and Hansinger alleges, according to the same authority, that "they have a polarizing membrane, and consequently electrical relations to the polarized vesicles of marsh mist, imbued with moisture, enriched by the humid exhalations, and screened by the shadows of night. These may form the most fruitful floating soil, for the invisible cells of the cryptogami, so that from the damp earth, or the nebulas air, or both, may come out to propagate disease, the cells of an anomalous vegetation."

Dr. Mitchell says again: "That almost every mineral, however poisonous, supports a peculiar cryptogamous vegetation. * * * Fungi grow in everything." Haller declares the Ag Piperatus is poisonous in France, but esculent in Russia. Another kind, an intoxicating food in Siberia, becomes a deadly poison in the south of Europe. The best varieties become unsafe after prolonged rains in France; and I have the authority of Col. Dobbins, of this city, for saying that they are poisonous to hogs in long wet seasons. Boiling water fails to destroy them: the acids and caustic ammonia are equally impotent to do so; and they sustain the cold of solid carbonic acid without abating their productive power, (*Cognare de la tour*).

But there is still another phase of cryptogami, if possible, more interesting than the history of the plants just given. The mould, which all have, perhaps, at some time of their lives, observed upon the walls of the house, all articles of leather, and sometimes the furniture, and even clothing, is of the class of plants we have been considering, and is a phenomena of ill omen to those in contact with it. It has been known to herald the advent of epidemics and endemics of the most fatal character.

Dr. Mitchell says: "Not only are common moulds more common on such occasions, but there often appears new and unusual productions of this kind. These moulds are chiefly red, but sometimes white, yellow, gray, or even black.

They appear, in an incredibly short space of time, on the roofs of the houses, on the pavements, on the clothes, veils, and handkerchiefs of women, on wooden and domestic utensils, and the meats in the larder. Even the depths of cellars, and the inmost recesses of chests and cupboards are invaded by a blood-like mould, which filled the observers with disgust and horror."

The presence of mould, and the rapid decomposition of vegetable and animal substances, the vast increase of insect life, seems to have a common cause in epidemic meteoration (Smith). Cragie observed this tendency in vegetable and animal matters to immediate decomposition, and attributed it to a "febriferous state of the atmosphere, displaying its insolubrity, not only upon the human race, but on the vegetable world, and dead animal and vegetable matter."

Plutarch, in his life of Romulus, says: "During the great plague at Rome, it seemed to rain blood." In the sixteenth century, during the great sweating sickness that prevailed over the continent, there appeared a blood rain at Cremoniæ. During a like disease in England, near the same time, the lowest order of cryptogami went hand in hand with the pestilence. A mould, like unto turbid dew, was cast upon every object, in the third century, having the appearance of gore, attended by a plague that swept off half the human race in fifteen years—(Eusebeus). In 1813, in the island of Malta, on the 14th of March, light showers were experienced, attended with a reddish mould. In April, the plague commenced, which nearly depopulated the island. During the pestilence at Naples, in 1660, curious mould spots appeared on the garments. And in that of 746, at the same city, mould spots appeared in the form of a cross upon the clothes of the populace—(Boyle). In 1502, the pestilence drove the people from Brussels for a period of three months. Upon returning, they found the roofs of their houses covered with mould, (Webster).

During the pestilential season of 1795, in New York, cabbages and different kinds of fruits were destroyed by the mould. Cherries did not come to perfection; and apples fell a month before their time: and those which came to maturity could not be preserved—(Bailey). Webster says the air of New York produced astonishing effects in the way of mould. Garments were spotted in a single night, pavements and utensils were covered, and carefully closed desks invaded by it. In Africa, the rains and sickness commence together; and the mould is so fearfully developed, as to rot the clothes and shoes in one or two days. In 1823 and 1825, during the epidemics of yellow fever in Natchez, Dr. Cartwright noticed an extraordinary mould: shoemakers complained that their leather and materials rotted, although there was no unusual dampness in the atmosphere, (Mitchell). In 1832, during the prevalence of cholera in Philadelphia, Dr. Mitchell noticed a splendid vermillion colored mucor, that covered paste, starch, and other vegetable preparations, never observed before or since.

I have witnessed two epidemics of erysipelas. In the fall of 1848, in the county of Crittenden, Ky., the leaves and grass became parched a month before the usual time, fruit fell, rotting from the trees, and vegetables perished in the garden. In November, a grayish dirty mold made its appearance on clothing and wooden articles, in the houses of the village and the surrounding country, which continued all winter. Simultaneously with it, the erysipelas made its appearance, generally attacking the throat, but not unfrequently the nose, face, scalp, ears, toes, fingers, arms, hands, feet, or legs. The lungs, liver, spleen, and peritoneum were frequently the points of attack; and during four months, not an obstetrical case survived in the locality where the disease prevailed.

In phlegmonous cases, gangrene generally set in, in an hour or two after the attack. Death not unfrequently occurred in one or two days, from congestion, strangling, or mortification. Nearly one-fourth of the cases died. Recoveries were generally slow, and, in many instances, attended with painful abscesses and whitlow, and with loss of hair, and finger and toe nails. The disease continued the entire winter, during which time there was a great amount of rain, and very little snow or cold weather. I have reason to believe, that erysipelas, and certain forms of sweating fever, of a most distressing type, are not unfrequently produced by eating bread made from diseased wheat, rye, and corn. It is known, that all of these cereals, some one of which enter into the daily tood of the whole human race, are liable, under certain meterological conditions, not well understood, to visitations from an army of cryptogams. When once grain is poisoned by them, no culinary process can render the bread harmless. Yeast may increase the deleteriousness of such food, by imparting new life and a new existence to the inchoate plant.

The history of epizootics shows examples of destructive diseases brought upon the lower animals by mould.

The angina maligna, the milzbrand, both gangrenous diseases; the foot evil in horses, with a multitude of febrile and eruptive diseases, are all capable of being conveyed, not only to other animals of the lower grade, but to man, also. The malignant pustule of sheep and cattle so poisons the pelts of those animals, that even tanning them into leather does not destroy it. The wool of sheep thus diseased, although cleansed, and woven into cloth, still retains the poison, and is capable of reproducing the disease, (Mitchell, Badham, Bayer). There is scarcely a doubt, but that milk-sickness has its origin in cryptogamous poisoning. A species of the fungi has been known to produce symptoms in some of the lower animals analagous to trembles, as the disease in the lower animals is called.

Just at the point where the miasmatists have stopped their investigations of the subject of the causation of fever, Dr. Salisbury commences his. His discoveries consist in finding in the expectoration of those affected with chills, certain double, oblong, nucleated cells, never before described. In the atmosphere of the adjacent ague levels, he found like cells, and numerous other abnormal bodies.

By the aid of the microscope, he discovers in the morning expectoration of chill subjects, certain zoophitic (zoosporoid?) cells, animalcule bodies, diatones, (?) dismidæ (?) algoid cells and filiments, and spores. But the most constant bodies found, are the minute oblong cells, both single and aggregated, and consisting of a distinct nucleus, surrounded by smooth cell wall, with a highly clear and apparently empty space between the outside cell wall, and the nucleus.

He next procured a number of glass plates. These he placed horizontally, twelve inches above the stagnant water, on four pegs, after sundown, and upon returning in the morning, found the under surface covered with the condensed vapour. To this he applied the microscope, and discovered innumerable cells, but very few of them answering to the description of the double oblong cells, found in the saliva of chill patients. Upon examining the top of the glass, he found them in multitudes. These cells, he thinks, are not fungoid, but of the algoid type, and evidently belonging to the palmellæ. By an after contrivance, he collected great quantities of these cells from the atmosphere, at various hights, the maximum being over one hundred feet above the nidus at Memphis and Nashville, and from thirty-five to sixty in the State of Ohio. He thinks he has demonstrated the identity of the cells on the top of the glass plates and in the atmosphere with those found in the morning expectoration of chill subjects; and by inference, reaches the conclusion, that these cells have an affinity for the epithelial surfaces, which they reach by inhalation, and are swallowed with the saliva, food, and water. That their absorption by the skin and mucous surfaces brings them in direct contact with the epithelial cells spread over, and covering the entire body, both internally and externally, through all the avenues by which external bodies may enter the organism. The epithelial cells being the first tissues with which these poisonous bodies come in contact, and having to pass through them before they can enter the systemic circulation, and reach the vascular tissues, they are so deranged as to poison the products organized by them; and these in turn poison other tissues, including the ganglionic and cerebro-spinal systems.

The epithelial cells of the spleen, mesentery, and liver being the most largely engaged in organizing nutrient products, these glands are the most severely taxed, and are the first to suffer extensively from the poisonous palmella, and hence it is, that grave lesions so frequently exist in these organs.

The oblong nucleated cells were invariably absent in the mucous secretions of persons above the ague levels. He found diatoms, dismidea, fungoid spores, and animalcular bodies at nearly all elevations above the ague line, especially in the neighborhood of streams and damp high grounds. In passing to the pools and swampy grounds, where many of his first experiments were made, he had to cross a peat bog: while doing this, he noticed a peculiar dry, feverish sensation was always produced in the throat and fauces, often extending to the pulmonary mucous surfaces, and that his expectoration, after returning, for some time, uniformly contained large quantities of the nucleated oblong cells of the palmellæ, or ague variety. His attention was thus drawn to the partially dessicated surface of the bog, recently broken by the tread of cattle. On this surface, rendered prolific in palmellæ by being disturbed, he discovered a whitish mould, resembling an incrustation from some salt. Upon suspending the glass plates over this soil, he discovered the oblong palmellæ cells he was in pursuit of. On placing a fragment of this incrustation under the microscope, it was found to be made up of aggregate masses of minute cells, so uniformly met with in the expectoration of those exposed to the cool vapors of malarial levels. These cells were algoid, and emanated from plants of a palmelloid type, but of several species. Amongst the larger ones the mucidinus fungi. These experiments were made near the city of Lancaster, Ohio. South east of the city, along the canal, is a peaty bog of some extent, surrounded by low, humid bottoms. Those living on the edge of the bog are frequently subjects of ague, from May to November, but most prevalent in August and September. To determine at what elevation the bodies found on the under surface of the glass plates could be traced, a funnel, with a weather-cock on the top, to keep the open mouth to the wind, a glass plate in the rear of the small end, covered with a concentrated solution of the chloride of calcium, were arranged, and all so constructed as to turn together, on a pivot, before the wind. By this contrivance, all the bodies floating in the air were found on the smeared surface of the glass screen.

The general summary of this test was as follows: "1st. That the cryptogamic spores and other minute bodies are mainly elevated during the night; that they rise and are suspended in the cold damp exhalations from the soil, after the sun has set; and that they fall again to the earth soon after it rises.

"2d. That in the latitude of Ohio, these bodies seldom rise above from thirty-five to sixty feet above the low levels; that in the northern and central portions of the State, they rise from thirty-five to forty-five feet; and in the southern from forty to sixty feet.

"3d. That at Nashville and Memphis they rise from sixty to one hundred feet and more, above the surface.

"4th. That above the summit plane of the cool night exhalations, these bodies do not rise, and intermittents do not extend.

"5th. That the day air of malarial districts is quite free from these palmelloid spores, and from the causes that produce intermittents."

These experiments are given in the author's own words: they are too important and too interesting to abridge.

With the view of testing more thoroughly and more carefully the local fever produced in the mouth, fauces, throat, and lungs, by inhaling the cells and sporoid bodies emanating from the vegetable organisms, on the freshly exposed, drying ground, he visited, Sept. 2d, 1862, "the bog referred to, for the purpose of collecting samples for further microscopic study. In a few minutes after my arrival on the bog, I began to feel a dry, feverish, constricted feeling in the mouth, fauces, and throat. The feeling increased till the fauces and throat became very unpleasantly parched and feverish. The opposite walls, in swallowing, adhered together, and the mucous secretions were quite entirely checked. There was a constant desire to swallow, hawk, and spit, without being able to raise much, or to relieve in the least, the dry, feverish, constricted sensation.

"The feeling soon extended to the bronchial and pulmonary surfaces, which became dry, feverish, and constricted, with a heavy congested sensation, and dull pain. These peculiar symptoms lasted about two hours after leaving the bog, before they entirely disappeared. The malarial matters inhaled, appeared to be poisonous to the surfaces with which they came in contact, and there seemed to be an effort on the part of the exposed mucous surfaces to close up their absorbent secretory organs, until this poisonous matter could be dislodged by the swallowing, and hawking and spitting, which they excited."

Twice afterwards he visited the same locality, and with the same result. The palmelloid cells are the only constant foreign bodies found in the spittle of those immersed in the cool night air of the ague levels. The source of these cells was found to be the palmelloid plants, growing in such profusion on the drying soils of ague lands during the prevalence of intermittents; and it is hence inferred, that the minute cell emanations from these low vegetable organisms are capable of exciting local fever in the mucous surfaces with which they come in immediate contact; and further, that there is strong presumptive evidence, from the details above given, that, by repeated and continued exposure to them, they may cause general fever, of either an intermittent or remittent type.

But all difficulties on the subject seem to approach a solution, in view of what follows: North of Lancaster, near an old canal mill, where the people are universally subject to chills, in a wide, low, rich prairie, a few rods south of the mill, and west of it, he found the ague palmella growing luxuriently, covering the soil recently thrown up by moles, and rendered fresh by the tread of cattle. In fact, all over the prairie, where the surface was broken up by any cause, the plants were developing in profusion. On the north edge of Lancaster, and on the west and south sides of Mount Pleasant, is another locality where ague prevails in its worst form, often running into fever of a remittent and continued type. There is a low belt of ground running through this locality, along which are stagnant pools of water. Around these pools, and in the rich, humid, broken soil, he found the ague palmella growing in profusion. On a healthy locality, where the chills had never occurred before, a healthy young man was attacked. He visited him with the attending physician. About fifteen rods south of the house was a new ditch, ten rods long, running through a piece of low, black, humid loam. The freshly thrown out earth and sides of the ditch were covered with ague palmella. Five miles northwest of Lancaster, at a place previously free from ague, he visited a family attacked with a severe form of the tertian type of intermittent fever. The gentleman was attacked on the first, and his wife on the third September. They were relieved by quinine, but relapsed on the 15th. This was arrested after the second paroxysm. Their dwelling stands on the edge of a low terrace, about thirty feet above the low bottom, which approached within fifteen rods of it, on the south and south-west sides. A small creek empties into the canal, about fifty rods from the house, which made a troublesome bar across the canal: to obviate this, a basin was excavated to receive the drainage of the creek. The soil thrown up was rich peaty loam, with black and blue clay. Before the work was completed, nearly all the hands were taken down with ague. At the time of his visit, about twenty days after the first case occurred, he found the newly excavated soil covered with ague plants. The gentleman and his wife slept in one of the lower rooms on the side of the house next to the excavation, whilst their children, seven in number, slept in the upper story, immediately over them. He and his wife were attacked on the 1st and 3d of September, whilst all his children escaped. The reason given is, that the fog from the newly excavated reservoir extended to the house, and about half way up the wall of the first story, and into his apartment, through the open window, bringing with it the same odour as the soil containing the ague plants, and produced the same febrile symptoms in the throat and fauces. The fog did not rise to the second story window, where the children slept, and disappeared before they were up. He had lived there forty years, and never before had chills in his family. He mentions this case to show that there is a fixed summit plane of invasion, above which the malarious causes do not extend. The best portion of the city of Lancaster stands on a hill, having an area of about one hundred acres: it rises to the hight of about sixty feet. The heavy, cold, night vapors, from the bogs adjacent, rise within fifteen feet of its summit. This marks distinctly the ague line: above this line chills do not occur, whilst all below are subject to it. So well marked is the ague line, that persons living in the lower story of a house, below the ague line, are all down at once with chills, whilst the inmates of the upper story, above the line, are exempt. Many localities were visited where intermittent fevers prevailed; and in every instance, without a solitary exception, the ague plants were found growing in the immediate vicinity of the disease; and in no instance were they found where the disease did not occur. At another locality, in this neighborhood, is a mill-pond: in August and September the water became very low. The ague palmellæ made their appearance on the drying peaty mud in great abundance. From the time these plants appeared till the last of September the wind was in the south. On the north side of the pond there was no one living, and consequently no ague cases. Near the last of the month the wind shifted, and blew briskly from the north and west. About thirty poles from the pond, and thirty feet above it, on a hill side, lived a strong, healthy, laboring family, who had been, up to this time, entirely free from ague.

The wind blew over the pond, directly towards his house. On the fourth day, several members of his family were taken down with chills. The wind again suddenly changed to the south-east, blowing directly towards the toll-gate, forty poles distant, where a family resided, in which were four small children. This family had, up to this time, been exempt from the disease. On the third or fourth day, two of the children were attacked; and, soon after, the father. These are interesting instances of the transmission of malarial influence by the winds.

The Hon. Mr. Burge, one and a half miles from Lancaster, resides on an elevation of one hundred feet above the ague levels, and far removed from them. For sixty-five years his place has been exempt from ague and fever. There were several springs of pure, cold water on the farm: one of which formerly supplied a fish-pond of about ten square roads in size. This pond was drained several years ago, and grew up thickly with grass. About half of it was spaded up in July, and the ague palmella appeared on the first of August. A portion of these were white, and others red, giving the appearance of having been sprinkled over with a thin layer of brick dust. August the 8th, Mr. B. and wife, who slept on the lower floor, began to feel languid with loss of appetite, and pains in the limbs and back. On the 20th,

Mr. B. had a severe chill—the paroxism lasting about three hours. On the 23d, the farm hand and his wife were attacked. Their houses were respectively ten and fifteen roods from the fish-pond. Mr. B.'s house north, and the tenant's house south of it. Dr. Salisbury examined the patch on the 22d. It was perfectly covered with ague palmella. They appeared, as usual, upon the broken thrown up soil. Those on the dry prominences were white, whilst those on the smoother and damper portion were the color of brick dust: the whole surface having the appearance of being sprinkled over with brick dust and lime. No cases occurred among the members of either family sleeping in the upper stories. At his suggestion, the patch was covered with straw to the depth of six inches. On suspending the glass plates over the straw for several nights, he obtained no traces of palmelloid spores or plants. Before this was done, the plates were covered with them nightly. The disease yielded readily, and there was no return of it. This result Dr. S. attributes to the covering of the miasmatic surface with straw.

Where the soil is free from lime, and the water soft, the ague palmella is mostly white, or diverging to yellow and green, and intermittents comparatively free from congestive tendencies, the types better marked, the eliminating organs less liable to derangement, and the symptoms more readily yield to proper treatment. In limestone regions, where the water is hard, and the soil is calcarous, during the months of July, August, and September, the malarious portion of the soil becomes covered with pink, brick red, yellow, or greenish palmellæ. The brick red and greenish are most abundant. In such localities, intermittents are apt to assume a congestive type. The eliminating surfaces and organs (epidermic and mucus, and portæ and renal glands) become much deranged, and their functions partially suppressed. Oxaluric and often phosphoric states follow; and when this is the case, quinine, iron, and arsenic alone, or all combined, do but little good, and often harm.

The soils around Camp Denizen, wherever exposed by eve drains, ditches, etc., become, in July, August, September, and October, covered with a green cryptogamic vegetation, which, in places, is of an ink black color. This vegetation is composed mostly of confervoid filaments, which are frequently terminated, when matured, by sporangiœ (?). These sporangiœ are noticed when the vegetation has assumed an ink black color, with a metalic lustre. Mixed with these filaments, are numerous palmelloid plants of two species: the one green, and the other brick red. When mature, these plants, as they become dry, send off multitudes of spores, which are elevated in the night exhalations.

Dr. S. has made extensive examinations, and has never found a case of ague where he did not find the ague plants; and per contra, he never found the ague plants without finding intermittent or remittent fever; or both, in proportion to their extent and profusion. These plants are not confined to the dessicating peaty bogs and humid low grounds, but are common to the drying beds of creeks, pools, ponds, and ditches; and also to calcarous soils, and even sandy planes in humid localities. These plants resemble, to the unaided eye, an incrustation of saline matter: this developes rapidly, as the soils dry; and as rapidly disintegrate and set at liberty their spores, which become elevated and suspended in the cold heavy night exhalations. These spores, as before stated, rise, usually, so that their upper surface in the northern and western States is marked by a plane varying from thirty-five to sixty feet above the ague grounds. The upper surface of these exhalations describes a horizontal plane, stretching away from the place of origin, in the direction traced by the wind. The spores and cells of these palmallæ are found diffused throughout these vapors, but do not extend above them. They occur, however, more abundantly at and near their upper surface than lower down. This will explain the singular fact, often noticed, that at a certain distance from the ague bottom, along the hill side, malarious diseases are frequently worse than on the bottoms themselves. The zone occupied by these exhalations has a temperature and hygrometric condition of its own, differing materially from the stratum of atmosphere resting immediately upon it, which is much warmer and dryer.

For the purpose of testing more fully, and, if possible, reaching results that would forever settle the causation of intermittent fever, he procured six boxes, and filled them with the malarious soil of a drying prairie bog, which was filled with palmellæ. These he removed five miles, to a locality where intermittent fever had never occurred. They were placed in the window of a room, in the second story of the house, in which two young men slept. On the sixth day, they both felt pains in the back and limbs, with loss of appetite, etc.: on the twelfth day, one was attacked with chill; and on the fourteenth, the other one was taken. Four members of the family were sleeping on the lower floor, none of whom were affected.

The experiment was repeated at another locality of equal healthfulness. Two boys and a man occupied an upper room. The two boys were taken down, one on the tenth, and the other on the thirteenth day. The man escaped. He could not obtain the consent of parties for further experiments. Dr. S. regards the experiments here made as eminently successful, and such must be the decision of all impartial minds. If his discoveries are confirmed by future observers, the result will be a final settlement of the puzzling question of the production of intermittent fever by marsh air. The benefit alike to science and humanity can hardly be estimated. To the science of medicine, especially, is this true; because the charge has often been made, that it was founded upon conjecture, and that its votaries had built few substantial monuments to perpetuate the fame of its discoveries in the field of occult causation. No class of men have labored more earnestly, or developed ideas more abundantly from the dark arcana of nature, than the brotherhood of medicine have done.

Discovery is a work of time. The field embraces all the elements of disease, distinct and general. The human system is a labarynth not to be learned in a life-time, but requiring many generations to unfold its secrets, and, one by one, to trace out its organs and functions, and the physical signs indicated in the opposite states of health and disease. Countless obstacles have been thrown in the road: numerous laws have been passed to protect graveyards, and top unish depredations upon them; and, at the same time, there are laws to punish physicians for mal-practice in medicine and surgery. The charge of ignorance and incompetency is frequently made, and results are pointed to in evidence of it: and all this in the face of existing laws that prevent and override inquiry. The physician is expected to understand the pathological conditions of the human system, and unravel the mysteries of causation by feeling of the pulse and looking at the tongue. If a post mortem is requested, it is almost invariably denied; and opportunities only occur, when the poor, friendless wanderer falls dead in the street, or in some public charity, to prosecute the study of the pathology and treatment of diseases. We give our dead friends to the mould and the worm to revel in and destroy specimens of morbid anatomy, that might open a new and glorious page in the history of medicine, if opportunities for investigation were allowed.

---

